# Immunohistochemical typing of amyloid in fixed paraffin-embedded samples by an automatic procedure: Comparison with immunofluorescence data on fresh-frozen tissue

**DOI:** 10.1371/journal.pone.0256306

**Published:** 2021-08-24

**Authors:** Antonella Barreca, Emanuel Bottasso, Francesca Veneziano, Manuela Giarin, Alberto Nocifora, Nadia Martinetti, Angelo Attanasio, Luigi Biancone, Giulia Benevolo, Dario Roccatello, Paola Cassoni, Mauro G. Papotti, the Amyloidosis Group of the “Rete Interregionale Piemonte e Valle d’Aosta per le Malattie Rare”

**Affiliations:** 1 Pathology Unit, City of Health and Science Hospital, Turin, Italy; 2 Department of Medical Sciences, University of Turin, Turin, Italy; 3 Department of Oncology, University of Turin, Turin, Italy; 4 Division of Nephrology Dialysis and Transplantation, Città della Salute e della Scienza Hospital, Turin, Italy; 5 Division of Hematology, Città della Salute e della Scienza Hospital, Turin, Italy; 6 CMID, Coordinating Center of the Network for Rare Diseases of Piedmont and Aosta Valley, and Nephrology and Dialysis Unit (ERK-net Member), San Giovanni Bosco Hub Hospital and University of Turin, Turin, Italy; Universita degli Studi di Milano-Bicocca, ITALY

## Abstract

Amyloidosis comprises a spectrum of disorders characterized by the extracellular deposition of amorphous material, originating from an abnormal serum protein. The typing of amyloid into its many variants represents a pivotal step for a correct patient management. Several methods are currently used, including mass spectrometry, immunofluorescence, immunohistochemistry, and immunogold labeling. The aim of the present study was to investigate the accuracy and reliability of immunohistochemistry by means of a recently developed amyloid antibody panel applicable on fixed paraffin-embedded tissues in an automated platform. Patients with clinically and pathologically proven amyloidosis were divided into two cohorts: a pilot one, which included selected amyloidosis cases from 2009 to 2018, and a retrospective one (comprising all consecutive amyloidosis cases analyzed between November 2018 and May 2020). The above-referred panel of antibodies for amyloid classification was tested in all cases using an automated immunohistochemistry platform. When fresh-frozen material was available, immunofluorescence was also performed. Among 130 patients, a total of 143 samples from different organs was investigated. They corresponded to 51 patients from the pilot cohort and 79 ones from the retrospective cohort. In 82 cases (63%), fresh-frozen tissue was tested by immunofluorescence, serving to define amyloid subtype only in 30 of them (36.6%). On the contrary, the automated immunohistochemistry procedure using the above-referred new antibodies allowed to establish the amyloid type in all 130 cases (100%). These included: ALλ (n = 60, 46.2%), ATTR (n = 29, 22.3%), AA (n = 19, 14.6%), ALκ (n = 18, 13.8%), ALys (n = 2, 1.5%), and Aβ_2_M amyloidosis (n = 2, 1.5%). The present immunohistochemistry antibody panel represents a sensitive, reliable, fast, and low-cost method for amyloid typing. Since immunohistochemistry is available in most pathology laboratories, it may become the new gold standard for amyloidosis classification, either used alone or combined with mass spectrometry in selected cases.

## Introduction

Amyloidosis encompasses a wide range of systemic or localized disorders in humans caused by the extracellular deposition of insoluble fibrils under the form of “amyloid”, in some cases leading to organ dysfunction. These deposits form homogeneous eosinophilic agglomerates staining positive for Congo red dye and showing apple-green birefringence under polarized light because of their characteristic β-pleated sheet conformation [1]. Congo red staining on amyloid deposits also shows bright red appearance under ultraviolet light on fluorescent microscopy (the so-called “Congo red fluorescence”, CRF) [2]. CRF is particularly useful as a diagnostic approach in cases of quite small amyloid deposits in which green birefringence may be weak or nearly absent [2].

The origin of amyloid deposits is always an anomalous serum protein, the amyloid precursor. To date, 40 different proteins have been identified as being amyloidogenic in humans [3]. The most common type of amyloidosis in high-income countries is acquired systemic immunoglobulin (Ig) light chain amyloidosis (AL), which results from the deposition of Ig light chains in the setting of a monoclonal plasma cell dyscrasia or a lymphoproliferative neoplasia. Other amyloidosis variants are related to chronic inflammatory diseases, like serum amyloid A protein amyloidosis (AA), or have a genetic background, being caused by hereditary or sporadic mutations in different genes encoding for soluble proteins, such as transthyretin amyloidosis (ATTR), fibrinogen amyloidosis (AFib), apolipoprotein A1 amyloidosis (AApo A1), gelsolin amyloidosis (AGel), cystatin C amyloidosis (ACys), and lysozyme amyloidosis (ALys), among others.

Several forms of amyloidosis present overlapping clinical manifestations, rendering differentiation on the sole basis of their clinical features quite difficult. However, an accurate and precise amyloid typing is crucial for therapeutic decisions: different forms of amyloidosis require different therapies, ranging from chemotherapy for AL amyloidosis to new pharmacological approaches for ATTR amyloidosis such as inotersen [4] and patisiran [5].

The diagnosis of amyloidosis is based on the histological detection of amyloid deposits in organs suspected of involvement or from sites commonly affected by amyloidosis in systemic forms. A second step includes the identification of the amyloidogenic protein. Various approaches for amyloid typing are currently used: immunofluorescence (IF) on fresh-frozen cryostat sections, immunohistochemistry (IHC) on fixed paraffin-embedded (FPE) tissues, immunogold labeling (IGL) on transmission electron microscopy (TEM) ultrathin sections, and laser microdissection (LMD) followed by mass spectrometry-based proteomics (MS). Even though LMD along with MS is currently considered the most reliable method for amyloid typing purposes, it represents a complex and expensive procedure that is not usually available in most laboratories [6]. While the debate regarding the gold standard method for amyloid typing is still ongoing, an IHC antibody panel to different amyloid proteins has recently been shown to be a sensitive and reliable tool for this purpose [7,8].

Therefore, the aim of this study was to evaluate the accuracy of this antibody panel using a standardized procedure in an automated IHC platform. Here we show that all cases of our present series were successfully subtyped and correlated with the available data from IF on cryostat sections.

## Methods

### Sample selection and data collection

Samples from different organs—including biopsies and surgical specimens—from patients with confirmed amyloidosis were retrieved and selected from the Pathology Unit records of the Città della Salute e della Scienza Hospital of Torino. Amyloidosis diagnosis was based on the presence of Congo red dye-positive deposits on light microscopy (LM), showing apple-green birefringence under cross-polarized light (GR), along with CRF.

Before this study was carried out, IF was the sole method normally used in our Institution in order to differentiate AL from AA amyloidosis whenever fresh-frozen material was available. This study consisted of two different patient cohorts: a retrospective one (comprising all consecutive amyloidosis cases analyzed between November 2018 and May 2020), and a “pilot” one, that included selected amyloidosis samples from 2009 to 2018. The latter cohort was made up of selected cases, enriched by those in which amyloid typing could not be achieved by IF, as well as other cases in which typing was carried out by means of IF. This “pilot” cohort of amyloidosis cases was initially used to set up the newly acquired panel of IHC antibodies. In particular, amyloidosis cases from this cohort that had been already characterized by means of IF enabled us to compare such results with those obtained by incorporating the new immunohistochemical method. In most cases complete clinical and/or laboratory and/or genetic data was available for which IHC results were compared to these data. In a minority of cases, IHC results could not be compared to these data since they were unavailable, or incomplete. No sample was collected specifically for the purposes of the research.

The specimens taken from organs other than kidneys were formalin-fixed and paraffin-embedded (FFPE). Conversely, kidney biopsies were alcohol-formalin-acetic acid (AFA)-fixed and paraffin-embedded (AFA-FPE). Compared to formalin, AFA fixative confers much better morphology to renal biopsies and similar antigen preservation [9]. In a few cases, bone marrow biopsies and a tibial spongy bone tissue sample were treated with a decalcifying solution (MicroDec EDTA-based, DiaPath, Bergamo, Italy) for 24–72 hours. Except for consultation cases from outside hospitals, the fixation time was strictly controlled to avoid under- or over-fixation (24 hours for formalin-fixed samples and 2 hours for AFA-fixed samples). Finally, in 82 cases fresh-frozen material was also available for IF.

Before carrying out the IHC tests, all materials and patient data were anonymized by a pathology staff member not involved in the study. None of the researchers had access to information that could identify individual participants during or after data collection. Fully anonymized data of patients’ medical records were analyzed from December 2020 to January 2021. The study was conducted according to the principles set out in the Declaration of Helsinki and was approved by the Research Ethics Committee of the University of Turin (DSM-ChBU; approval number: 07/2020). All the relevant data are within the article. The remaining data are available from the corresponding author upon reasonable request.

### Immunohistochemistry (IHC)

Three μm thick sections were cut from FFPE or AFA-FPE samples and collected on positively charged slides. IHC was entirely performed in a BenchMark ULTRA automated stainer (Roche Ventana Diagnostics, Oro Valley, USA). The following primary antibodies (amY-kit, amYmed, Munich, Germany) were used: anti-AA (mcC clone), anti-ALλ (HAR), anti-ALλ (ULI/LAT), anti-ALκ (SIN/GAT), anti-ALκ (KRA/KUN), anti-ATTR (TIE), anti-AHγ (SOL/mix), anti-Aβ_2_M (WOE), anti-SAA_4_/cSAA, anti-AFib, anti-AApoAI, anti-ALys, anti-ACys and anti-AGel, and anti-AA (red clone, kindly gifted by Prof. Reinhold P. Linke). All antibodies were polyclonal, except for the monoclonal anti-AA antibodies. The development and validation of this panel of antibodies were reported by Linke [7]. The IHC protocol originally designed for manual indirect immunoperoxidase procedure was here adapted for the automated Ventana Roche IHC platform. In detail, prior to immune reactions, antigen retrieval was performed with CC1 solution at 92°C for 36 minutes. The primary antibody incubation step had a two-hour duration for all of them. Conversely, each antibody was set at the most suitable dilution ranging from 1/5 up to 1/150. Finally, in place of the reported peroxidase-antiperoxidase method [7], the reaction was developed with the ultraView Universal DAB detection kit (Ventana Roche), according to the manufacturer’s instructions. In particular, this detection biotin-free kit consists of an anti-mouse and anti-rabbit Ig secondary antibody directly conjugated to a horseradish peroxidase polymer. This polymer-based signal amplification method reduces the amount of primary antibody needed, shortens the incubation period, and reduces the background, thus optimizing the quality of the reaction.

As previously described by Linke [7], the interpretation of IHC results with this panel of antibodies is based on a comparative evaluation both from the strength and the extension of the reactions. As a matter of fact, it is crucial to identify the most uniform and strongest positivity (diagnostic reactivity, equally distributed all over the amyloid deposits) from the inconsistent unspecific staining (weak, non-uniform, and not widespread reactivity within the amyloid deposits).

### Immunofluorescence (IF)

Three μm thick sections were cut with a cryostat from fresh-frozen samples. They were collected on positively charged slides and fixed for 10 minutes in cold acetone. For direct immunofluorescence, sections were incubated with the following FITC-conjugated antibodies: anti-human Kappa Light Chain-FITC (Diagnostic BioSystems, Pleasanton, CA, USA), anti-human Lambda Light Chain-FITC (Diagnostic BioSystems), anti-human Kappa Light Chain-FITC (Cytognos, Salamanca, Spain), and anti-human Lambda Light Chain-FITC (Cytognos). For indirect immunofluorescence, sections were first incubated with the following primary antibodies: anti-human Amyloid A (mc1 clone, Dako Agilent, Santa Clara, USA), and anti-human P Component (Dako). Sections were then incubated accordingly, with the appropriate secondary FITC-conjugated antibodies: anti-rabbit IgG-FITC (Southern Biotech, Birmingham, USA), and anti-mouse Ig-FITC (Southern Biotech).

### Statistical analysis

Comparisons between groups were performed by non-parametric methods, such as the Mann-Whitney U test for quantitative variables, whereas the chi-square test was employed when dealing with categorical variables. Data were considered statistically significant if p <0.05.

## Results

### Pathological characteristics

A total of 143 amyloid samples from 130 patients were included. One hundred and nineteen patients underwent only one biopsy, whereas 9 and 2 patients underwent 2 and 3 biopsies, respectively. In most cases, a repeat biopsy was performed to evaluate the extent of amyloid deposition, whilst in two cases a second biopsy was done due to inconclusive IHC results of the first one.

The “pilot” cohort was composed of 51 patients (39%), with a mean age (± SD) of 66.9 ± 11.8 years (range 36–86) (Table 1). Thirty-five patients were males and 16 were females. This cohort was constituted by cases that still had an unclassified form of amyloidosis (40 patients, 78.4%), whereas in the remaining 11 patients (21.6%), amyloid typing had already been performed by means of IF alone and such results were compared to those obtained by IHC. The retrospective cohort was made up of 79 consecutive patients (61%), with a mean age (± SD) of 70.6 ± 10.3 years (range 42–87). Thirty-seven were males and 42 were females (Table 1). Both cohorts had no age-related differences whereas a slight difference in gender distribution between the two cohorts (p <0.025) was observed. However, to the best of our knowledge, sex is known to exert no influence on amyloidosis development in most of the amyloidosis forms presented here (AL, AA, ALys, and Aβ_2_M amyloidosis), with a recent report showing that ATTR amyloidosis may display a male gender preponderance [10]. Hence, the results from both study groups were analyzed and presented together.

**Table 1:** Amyloidosis cases characteristics.

	“Pilot” cohort	Retrospective cohort
**Total patients**	51	79
**Male**	35 [69%]	37 [47%]
**Female**	16 [31%]	42 [53%]
**Mean age ± SD**	66.9 ± 11.8	70.6 ± 10.3
**Age range**	36–86	42–87

Sample source included biopsies and surgical specimens from various organs and locations, as detailed in Table 2.

**Table 2:** Origin of biopsy samples.

Organ or location	N.	%
Salivary glands	35	24.5
Kidney	35	24.5
Periumbilical fat	19	13.3
Endomyocardium	10	6.9
Rectum	8	5.6
Stomach	7	4.9
Colon	4	2.8
Bone marrow	4	2.8
Liver	4	2.8
Lung	3^a^	2.1
Duodenum	2	1.4
Spleen	2^a^	1.4
Skin	2	1.4
Gallbladder	1	0.7
Omentum	1	0.7
Nasopharynx	1	0.7
Pleura	1	0.7
Tibial spongy bone	1	0.7
Cervical lymph node	1	0.7
Larynx	1	0.7
Carpal tunnel (fat tissue)	1	0.7
**Total samples**	**143**	**100**

^a^Two splenic and one pulmonary samples corresponded to surgical specimens.

### Immunohistochemistry (IHC) for amyloid characterization

In our series, the distribution of the various amyloidosis types by means of IHC is reported in Table 3, including ALλ (n = 60, 46.2%), ATTR (n = 29, 22.3%), AA (n = 19, 14.6%), ALκ (n = 18, 13.9%), ALys (n = 2, 1.5%), and β_2_ microglobulin amyloidosis (Aβ_2_M, n = 2, 1.5%).

**Table 3:** Amyloidosis typing.

Diagnosis	N. (%)	Mean age (±SD)	Age range
AL	78 (60.1)	70 ± 10	41–87
ALλ	60 (46.2)	70.5 ± 9,4	44–87
Alκ	18 (13.9)	68.2 ± 11.8	41–82
ATTR	29 (22.3)	72.6 ± 11	39–87
AA	19 (14.6)	64.2 ± 11.9	36–83
ALys	2 (1.5)	57 and 42 years old	
Aβ_2_M	2 (1.5)	Both aged 56 years	
Total patients	130 (100)		

These findings were obtained using a first round of four antibodies (anti-ALλ (ULI/LAT), anti-ALκ (KRA/KUN), anti-AA (red clone), and anti-ATTR (TIE) in 116 cases. In 10 cases (7.7%), two additional antibodies directed against light chains, namely anti-ALλ (HAR) and anti-ALκ (SIN/GAT), were also applied when unspecific staining of the uninvolved light chains was suspected. For those cases in which all reactions were found to be negative or inconsistent, the remaining 9 antibodies included in the panel were used: anti-AHγ (SOL/mix), anti-Aβ_2_M (WOE), anti-SAA_4_/cSAA, anti-AFib, anti-AApoAI, anti-ALys, anti-ACys, anti-AGel, and antiAA (mcC clone).

Following this approach, a highly accurate typing of the commonest forms of amyloidosis was achieved. Specifically, IHC allowed us to identify a single amyloid variant in all 130 cases from the series (100%). These IHC results turned out to be in full agreement with clinical, laboratory, and genetic data from the 124 patients in whom this information was available (95% of total cases). Of note, in 2 cases (1.5% of total cases), the characteristics of samples in the first biopsy (very small fragments of periumbilical fat tissue in one case and poorly-fixed spleen in the other one, which constituted a consultation case from an outside hospital), led to confusing results with unspecific positivity for anti-AFib antibody. In both cases, a second biopsy was then needed to reach the final diagnosis of ALλ amyloidosis in one case and ALκ amyloidosis in the other one.

Importantly, IHC enabled us to classify amyloid deposits in all cases in which IF proved to be ineffective (n = 44, 33.8% of all patients), hence allowing amyloid characterization in cases that by IF would have remained unsolved, requiring an alternative procedure for amyloid typing. On the other hand, in all cases that were successfully characterized by IF (n = 30, 23.1% of all patients), a 100% agreement with IHC results was observed.

### Characteristics of patients with the different amyloidosis variants

#### AL amyloidosis

Seventy-eight AL amyloidosis patients (60% of cases) were diagnosed at a mean age (±SD) of 70 ± 10 (range 41–87 years). Among them, 41 patients (52.6%) were males (mean age 69 ± 11.6, 41–87) and 37 (47.4%) were females (age 71.1 ± 7.9, 50–86). Sixty cases corresponded to an ALλ variant (76.9%, mean age 70.5 ± 9.4, range 44–87), and 18 to the ALκ one (23.1%, 68.2 ± 11.8, 41–82).

Complete clinical and/or laboratory data were not available for 4 patients (5.1%, 3 ALλ patients, and a single ALκ patient). Among the remaining 74 cases with available clinical and/or laboratory data, 72 patients (97.3%) had an underlying hematological condition, observed by a detectable blood serum and/or urinary paraprotein or elevated serum lambda or kappa chains with an abnormal ratio which in all cases coincided with the type of AL diagnosed by IHC (i.e. λ or κ). In the remaining 2 patients (2.7%), no monoclonal protein was detected, an event occurring in some cases, depending on the amount of serum paraprotein and the sensitivity of the method that is adopted [11,12].

**Fig 1:**
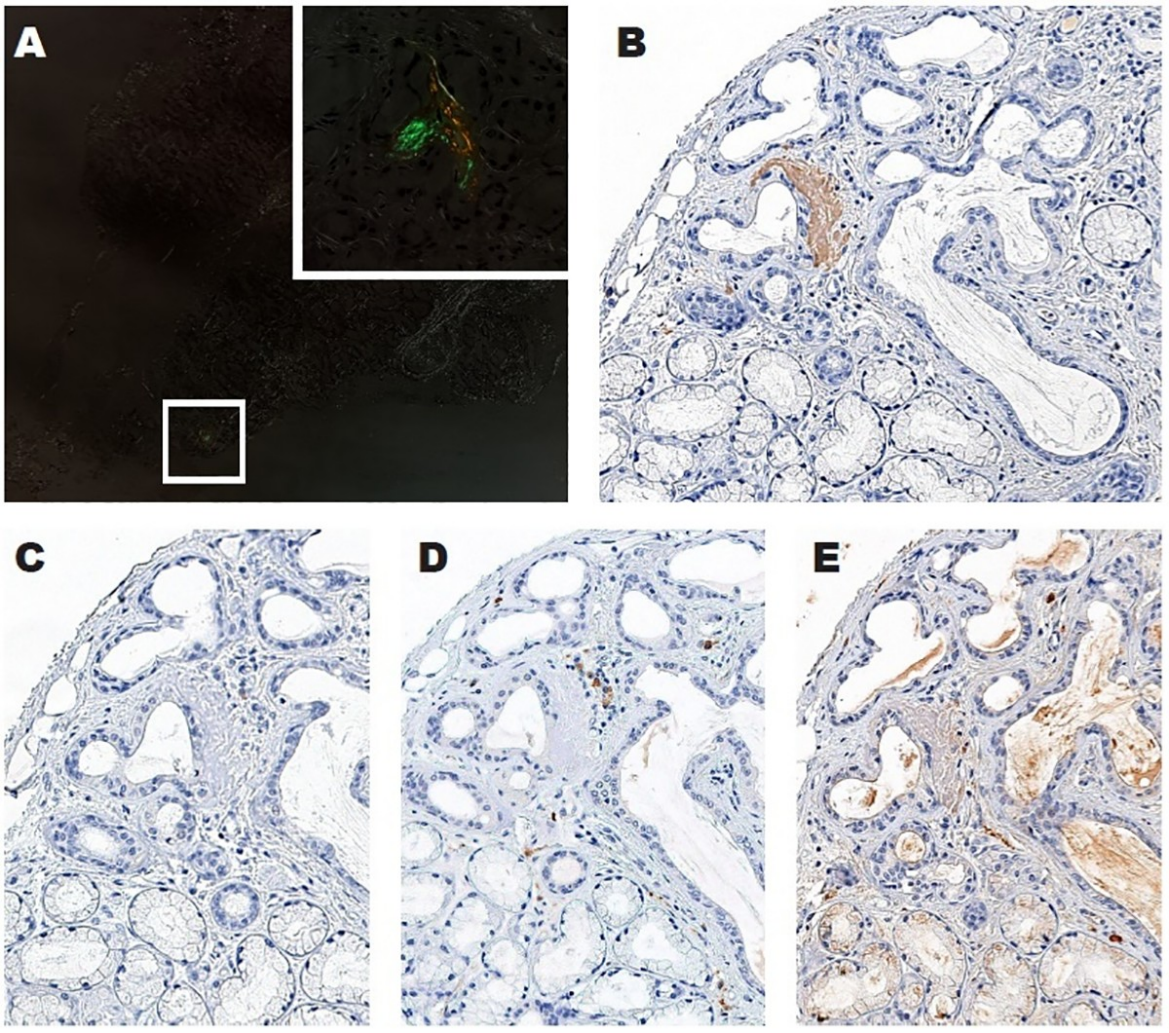
Amyloidosis diagnosis and characterization. In this case of ATTR amyloidosis, only a quite small deposit of amyloid was identified adjacent to a duct on a salivary gland biopsy, showing the typical apple-green birefringence under cross-polarized light following Congo red staining (A, x30; inset x400). The connective tissue exhibits an unspecific white refringence. IHC allowed the characterization of this deposit, which turned out to be intensely positive for anti-ATTR (B, x400). No significant consistent staining was observed within the amyloid deposit for anti-AA (C, x400), anti-ALκ (D, x400), and anti-ALλ (E, x400). As expected, these two latter antibodies were positive in stromal plasma cells, whereas anti-ALλ had a weak non-specific background outside the amyloid deposit.

**Fig 2:**
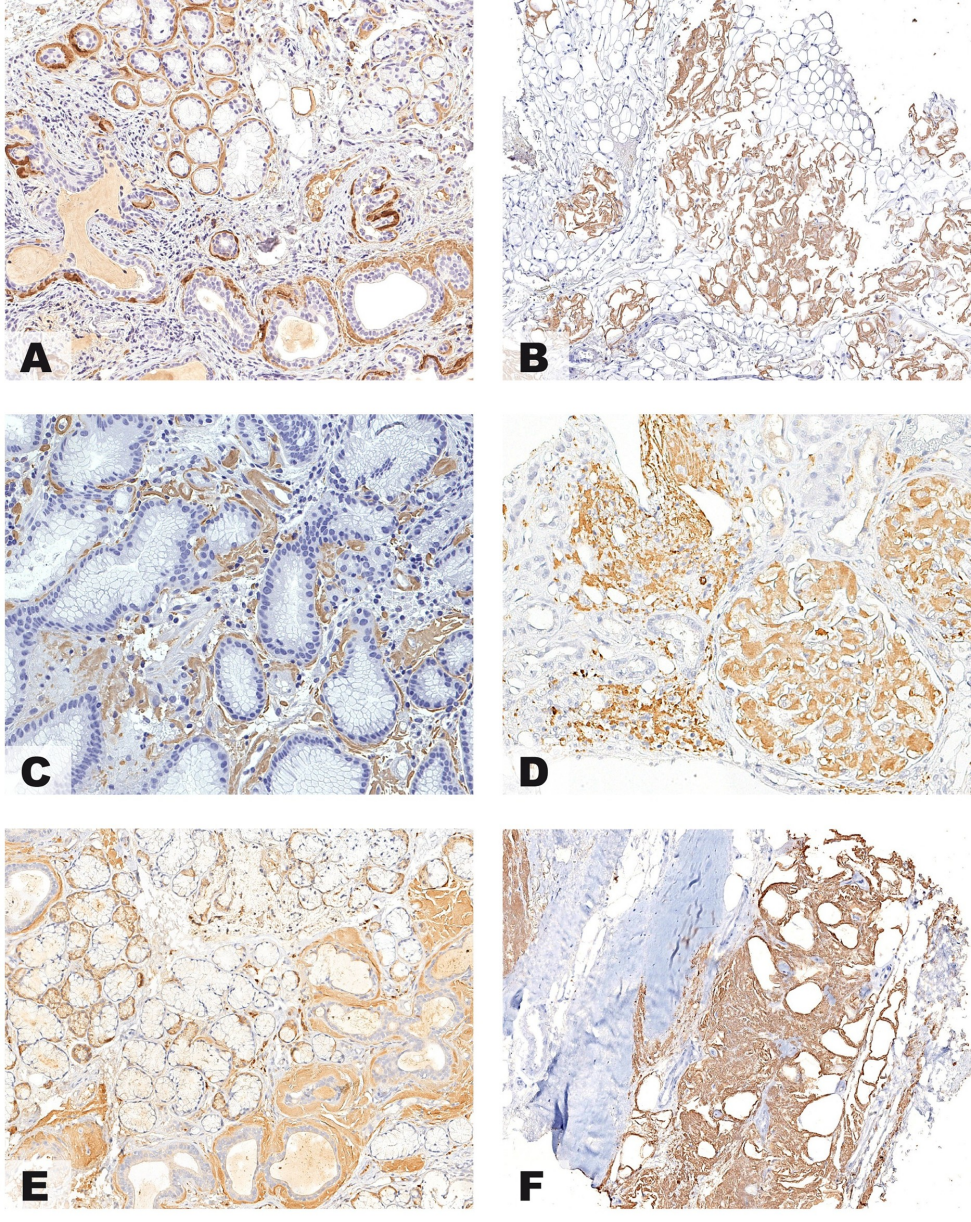
Immunohistochemistry of amyloid deposits. Minor salivary glands biopsy detected areas of amyloid substance in periductal and periacinar location that stained strongly only for anti-ALλ (ULI-LAT) (A, x200). Periumbilical fat biopsy in an 80-year-old male demonstrated a septal and vascular pattern of amyloid deposition that resulted brightly positive only for anti-ATTR (TIE) (B, x200). Antral gastric biopsy revealed interstitial and vascular amyloid deposits that were strongly positive only for anti-AA (red clone) (C, x200). Renal biopsy demonstrated glomerular and interstitial amyloid deposition with bright positivity for anti-ALk (KRA/KUN) (D, x200). Minor salivary glands biopsy detected, in this 42-year-old female, amyloid deposits located in the interstitium and around ducts which turned out to be intensely positive for anti-Alys (E, x200). In this case of Aβ_2_M amyloidosis, Congo Red staining demonstrated large deposits of amyloid in the extracapsular fibroadipose tissue of the transplanted kidney with strong positivity only for anti-Aβ_2_M (F, x200).

As expected [13], while anti-ALκ antibodies showed intense positivity in their specific amyloid deposits mostly without unspecific staining, a slight background was sometimes observed with anti-ALλ antibodies. In particular, in 6 out of 18 ALκ, 10 out of 29 ATTR, 13 out of 19 AA, and 1 out of 4 rare ALys and Aβ_2_M amyloidosis cases, a weak and irregularly distributed unspecific reactivity for anti-ALλ antisera was observed (Figs 1 and 2). Nevertheless, the use of two different anti-ALλ antibodies contributed to overcoming this issue and this unspecific staining turned out to be perfectly distinguishable from true, strong, and regularly distributed positivity within amyloid deposits (Fig 2).

#### ATTR amyloidosis

Patients with ATTR amyloidosis (n = 29, 22.3% of cases), had a mean age (±SD) of 72.6 ± 11 (range 39–87 years). Twenty-two patients (75.9%) were males (73 ± 9.2, 44–87 years) and 7 (24.1%) were females (71.3 ± 16.3, 39–84 years). As expected, IF was useless to characterize these cases. Conversely, IHC staining provided a specific signal in amyloid deposits of all cases in the absence of significant reactivity for any other antibody. This signal was clearly detectable even in cases with minimal amounts of amyloid accumulation (Figs 1 and 2).

Eight of these patients (27.6%) underwent genetic analysis, and a wild-type form was diagnosed in 7 of them (aged 68 ± 7.1, 60–79 years). A mutation was found in the remaining 39-year-old female patient, confirming a hereditary form. Genetic studies were not available for the remaining 21 cases (72.4%), being worth noticing that 12 of these patients belonged to the retrospective cohort and had an incomplete workup, including genetic tests.

Importantly, among patients with ATTR amyloidosis, 7 (24.1%, aged 77.8 ± 5.1, 70–84 years at the time of amyloidosis diagnosis) presented a detectable monoclonal paraprotein for which an AL form was clinically suspected. As it is well known, monoclonal gammopathy is quite frequent in people older than 50 years of age [14] and these paraproteins are not always amyloidogenic. Hence, caution should be exerted in these cases to avoid AL misdiagnosis and possibly harmful consequences [15].

#### AA amyloidosis

The 19 AA amyloidosis patients (14.6% of cases) had a mean age (± SD) of 64.2 ± 11.9, 36–83 years. Six of them were males (56.3 ± 12.7, 36–70 years), and 13 females (67.8 ± 9.9, 58–83 years).

A history of chronic inflammatory disease or chronic infection was present in 16 of them (84.2%), which included rheumatoid arthritis or familial Mediterranean fever among the most common ones. The other three patients (15.8%) had no known history of chronic inflammation at the time of AA amyloidosis diagnosis. IHC using the monoclonal anti-AA antibody (red clone) was positive in all cases of this group and largely overlapped the profile detected by conventional IF. This antibody showed no unspecific staining in all the remaining amyloidosis cases different from AA (Figs 1 and 2).

#### ALys and Aβ_2_M amyloidosis

As for ATTR amyloidosis, IF was useless in these cases, with IHC being critical for case definition. Altogether, 2 cases of ALys amyloidosis (1.5% of total cases) and 2 cases of Aβ_2_M amyloidosis (1.5% of total cases) were identified.

ALys amyloidosis cases included a 57-year-old male and a 42-year-old female. The male patient’s mother died of suspected, non-histologically proven, intestinal amyloidosis. Following our diagnosis, he underwent genetic studies confirming an ALys form. In the case of the female patient, genetic studies are still pending, whereas a history of endometriosis and abdominal pain was reported.

Both Aβ_2_M amyloidosis cases were males, aged 56 years. They had a history of long-standing chronic renal failure, hemodialysis, and multiple kidney transplants with subsequent graft loss.

IHC with anti-ALys and anti-Aβ_2_M antibodies proved to be successful in all tested cases, showing a clear and intense positivity in the specific amyloid deposits, with no significant tissue background.

#### Other uncommon amyloidosis variants

No uncommon types of amyloidosis were identified in the current series using the other antibodies developed to the rare amyloid proteins associated with such variants.

### Immunofluorescence (IF) in amyloid typing

Prior to this study, IF on fresh-frozen tissue was the sole method for amyloid typing in our laboratory. A simple panel of four antibodies was used for this purpose, allowing characterization into three categories: ALλ, ALκ, and AA amyloidosis.

In the current series of 130 cases, fresh-frozen tissue was available for 82 (63%). Forty-two of them (51.2%) belonged to the retrospective cohort, whereas the remaining subjects (n = 40, 48.8%) were part of the “pilot” cohort.

Apart from eight cases with no amyloid deposits in the submitted material (9.8%), IF was successful in only 30 cases (36.6%), diagnosed as ALλ (n = 16, 53.3%), AA (n = 9, 30%), and ALκ (n = 5, 16.7%) amyloidosis. Notably, in the remaining 44 patients (53.7% of cases), IF failed to identify a specific type of amyloid deposit or provided inconclusive results. In particular, amyloid deposits turned out to be reactive for both light chains with no clear predominance of either, or were positive for anti-AA antibody and simultaneously for one or both light chains, or, finally, were only positive for the P Component of amyloid, being negative for the remaining reagents.

### Comparison of IHC and IF data

The 82 cases in which fresh-frozen material was available were tested by both IHC and IF. The results showed a complete overlap of the two procedures in all the 30 previously referred cases in which IF provided conclusive findings. Importantly, all the remaining 44 inconclusive IF cases were successfully characterized through IHC with the mentioned panel of antibodies, with the identification of the following amyloid types: ATTR (n = 19), ALλ (n = 11), ALκ (n = 6), AA (n = 5), ALys (n = 2,) and Aβ_2_M (n = 1).

## Discussion

In this study, we report that an automated and standardized IHC procedure for 15 commercial antibodies to various amyloid proteins proved to be a sensitive, reliable, and low-cost tool for amyloid typing in fixed and paraffin-embedded tissues. Through this method, a specific amyloid form was identified in every case from the present series. Importantly, these IHC results were in complete agreement with IF findings, as well as clinical, laboratory, and genetic assessment, in all cases in which these data were available.

In only two cases (1.5%), the characteristics of the samples from the first biopsy led to confusing results because of the unspecific anti-AFib antibody positivity, thus requiring a second biopsy for a definitive diagnosis. Importantly, not only did IHC enable amyloid typing in formalin-fixed samples, but it also made it possible on the alcohol-formalin-acetic acid (AFA)-fixed ones (the fixative we normally use in our Institute for kidney biopsies), as well as on a few decalcifying solution-treated samples (bone marrow biopsies and a tibial spongy bone tissue specimen). Therefore, almost any type of material can be examined and typed by this IHC procedure.

As stated above, prior to this study, IF on unfixed frozen specimens was usually used in our laboratory to distinguish AL forms from AA amyloidosis. In this series, IF proved to be useful for amyloidosis subtyping in only 30 out of 82 patients for whom fresh-frozen tissue was available (36.6% of cases), all fully overlapping with IHC results (100% of these cases). In 44 cases (53.7%) with amyloid deposits, IF on fresh-frozen tissue failed to provide a definitive classification due to simultaneous positivity for both light chains with no predominance of either of them, combined reactivity for anti-AA antibody and anti-λ and/or anti-κ antibodies, or positivity only for the P Component of amyloid and negativity for the remaining reagents. Remarkably, the newly introduced immunohistochemical approach allowed to define the subtype of all such 44 cases, which would have otherwise required more sophisticated and expensive subtyping techniques. Among these, several ATTR amyloidosis cases were identified (n = 19). These patients may take advantage of specific novel treatments and a fast and reliable procedure directly applied on FPE tissue sections which not only may help to save time but also to reduce diagnostic cost [4,5].

The current findings agree with earlier studies that reported a high diagnostic accuracy of IHC on FPE tissue for amyloidosis typing. Linke et al. [16] developed and characterized the panel of antibodies used in our study, though with no correlative analysis with IF and using a manual procedure. The authors demonstrated a 97.9% sensitivity and 99.3% specificity of IHC in 581 samples from previously unclassified amyloidosis cases through this panel. Using part of this same antibody panel (10 antibodies in total), Lassner et al. [17] were able to define the amyloidosis type in all the 25 cases in which sufficient tissue for immunohistochemical analysis was available in a series of 31 endomyocardial biopsies containing amyloid deposits. Similarly, Schönland et al. [18] reported that IHC was able to define the amyloid type in 94% of cases from 117 patients, studied in parallel by clinical examination, laboratory tests, and genotyping. In this latter study, a different panel composed of 10 antibodies was employed. Thus, while other studies have shown the reliability of IHC as a method for amyloid characterization, to the best of our knowledge, this is the first study in which this panel of antibodies has been tested by a completely automatized procedure. Moreover, in our work, IHC results have been compared with IF findings, along with clinical, laboratory, and genetic assessment, in all cases wherein these data were available.

The prevalence of the observed various amyloid types agrees with reported data in Western European countries [16–19]. The combined AL amyloid isotypes are the commonest type of amyloidosis in most series (with a clear higher incidence of ALλ *versus* ALκ forms), followed by ATTR amyloidosis cases, with AA amyloidosis apparently showing a decline in the number of cases [20,21]. The current series has a higher percentage of ATTR amyloidosis cases, probably since almost half of them belonged to the “pilot” series, which was enriched by cases of amyloid deposits unsolved by IF tests. Other groups referred to the lack of reliability of IF in distinguishing AL from AA forms when applied to renal biopsies, extending these conclusions to IHC, which in their opinion may reveal equivocal results in a significant number of cases [22,23]. In fact, it is well recognized that, beyond specialized centers, and without the use of a validated panel of antibodies, IHC may lead to confusing results due to unacceptably high background staining [13].

In this regard, a particularly well-known IHC problem in characterizing amyloid deposits is the non-specific entrapment of light chain antibodies. In fact, unspecific staining for Ig light chains (mainly related to λ-light chain), as well as false-negative results for Ig light chains with some commercial antibodies in cases of AL amyloidosis, have long been a well-recognized problem in IHC reactions [13,19,24]. Multiple factors account for this issue, such as conformational differences between serum versus tissue-fixed Ig light chains, antigen retrieval methods, heterogeneity of Ig light chains because of their conspicuous variable domains, mutations of Ig light chain epitopes that are usually recognized by the available antibodies, Ig light chain fragmentation during amyloid fibrillogenesis, and quality of some commercial antibodies [23–27]. In this regard, it is worth noting that whereas similar limitations were also observed in our series with IF, as previously stated, the newly introduced immunohistochemical approach for amyloidosis subtyping overcame these inconsistencies, thus rendering the typification quite straightforward in most cases. Specifically, the use of the mentioned panel of antibodies, comprising two different anti-λ light chain and two different anti-κ light chain reagents for IHC on FPE samples, along with an accurate standardized automated technical procedure, mostly eliminated confusing results. Therefore, even though a slight amount of unspecific background still remains (particularly for anti-ALλ and anti-AFib antibodies), it poses no major diagnostic concerns or pitfalls, becoming perfectly discernible from true positivity by a specialized pathologist in the field (see Figs 1 and 2).

Coming to the point of the gold standard approach for amyloid typing, a general belief states that immunogold labeling on TEM ultrathin sections is more reliable than light microscopy IHC on FPE samples [28,29]. In this regard, it is worth remembering that both techniques are based on the same principles, and the procedures are essentially equivalent, mainly differing on the detection system (i.e., enzymatic oxidation of a chromogen *versus* gold particles). Thus, in both methods, reliability rather lies in the quality and specificity of the employed antibodies, the accuracy of technical processing, and the expertise of the pathologist interpreting the results. In fact, IHC by LM and IGL by TEM have been shown to be equally reliable for amyloid deposit subtyping [7,30]. On the other hand, when compared with IGL, IHC is simpler, faster, less expensive, and almost universally available in diagnostic laboratories. In addition, when performed in automated platforms, it is highly standardized and reproducible. In the present study, minor modifications were introduced in the original protocol [7], mainly with the addition of an antigen retrieval step and a different signal amplification system, which resulted in cleaner and easier-to-interpret reactions. Finally, whereas IHC by LM allows the examination of relatively large tissue areas, IGL by TEM can only be applied to minuscule fragments of tissue. Thus, the selection of the minute piece of tissue to be examined by TEM is a crucial and risky step during the technical processing, potentially leading to false-negative results in the absence of amyloid deposits in the area submitted to ultrathin sectioning.

To date, mass spectrometry is still considered the most reliable and accurate method for identifying the exact protein present in amyloid deposits, and hence the amyloidosis variants [6,31–33]. In the opinion of most experts in the field, MS and IHC are both indispensable and complementary to one another [16,34]. In fact, in two international multicenter studies, similar specificity for IHC and MS in amyloid typing was detected, with a reported higher sensitivity of IHC when laser microdissection was not applied on samples prior to MS, and a similar sensitivity when it was applied [7,30]. IHC may also be more suitable for those cases in which only very tiny amyloid deposits are encountered. In these situations, with scarcely represented amyloid spots on tissue samples, MS cannot be performed, being immunohistochemical typing still possible on a whole section. However, it is worth noticing that IHC completely fails to identify the most recently described and infrequent forms of amyloidosis, due to the lack of commercially available specific antibodies for the particular amyloid protein. In these circumstances, as well as for the IHC inconclusive cases, LMD and MS remain essential for a definitive diagnosis.

Extending and confirming former demonstrations [7,8], our work shows that with the appropriate antibodies, a standardized automated technical procedure, and the expertise of a highly specialized pathologist in the field, IHC is a very accurate and reliable method for amyloid typing. Compared to IF, this new approach significantly improved accuracy in the identification of amyloid variants. Moreover, when compared to the other more sophisticated methods, IHC represents an easy and well-standardized procedure, when automated platforms are available, enabling amyloid typing with a fast and low-cost technique. However, it is worth noting that this antibody panel still needs further validation. In this sense, our future work will focus on performing LMD along with MS in this same series of cases for comparison purposes. In our view, an immunohistochemical screening on fixed paraffin-embedded samples, combined with MS in selected controversial cases, may represent the new gold standard for amyloidosis characterization.
